# Explorative Image Analysis of Methylene Blue Interactions with Gelatin in Polypropylene Nonwoven Fabric Membranes: A Potential Future Tool for the Characterization of the Diffusion Process

**DOI:** 10.3390/gels9110888

**Published:** 2023-11-09

**Authors:** Jan Zidek, Anna Sudakova, Jiri Smilek, Duc Anh Nguyen, Hung Le Ngoc, Le Minh Ha

**Affiliations:** 1 Central European Institute of Technology (CEITEC), Brno University of Technology, Purkynova 123, 612 00 Brno, Czech Republic; 2 Faculty of Chemistry, Brno University of Technology, Purkynova 464/118, 612 00 Brno, Czech Republic; 3 Center for Research and Technology Transfer (CRETECH), Vietnam Academy of Science and Technology (VAST), 18-Hoang Quoc Viet, Nghia Do, Cau Giay, Hanoi 100000, Vietnamngoc10hung@yahoo.com (H.L.N.); 4 Graduate University of Science and Technology (GUST), Vietnam Academy of Science and Technology (VAST), 18-Hoang Quoc Viet, Nghia Do, Cau Giay, Hanoi 100000, Vietnam; 5 Institute of Natural Products Chemistry (INPC), Vietnam Academy of Science and Technology (VAST), 18-Hoang Quoc Viet, Nghia Do, Cau Giay, Hanoi 100000, Vietnam; halm2vn@gmail.com

**Keywords:** methylene blue, gelatin, image analysis, dye binding, resonance states, layered materials

## Abstract

This manuscript explores the interaction between methylene blue dye and gelatin within a membrane using spectroscopy and image analysis. Emphasis is placed on methylene blue’s unique properties, specifically its ability to oscillate between two distinct resonance states, each with unique light absorption characteristics. Image analysis serves as a tool for examining dye diffusion and absorption. The results indicate a correlation between dye concentrations and membrane thickness. Thinner layers exhibit a consistent dye concentration, implying an even distribution of the dye during the diffusion process. However, thicker layers display varying concentrations at different edges, suggesting the establishment of a diffusion gradient. Moreover, the authors observe an increased concentration of gelatin at the peripheries rather than at the center, possibly due to the swelling of the dried sample and a potential water concentration gradient. The manuscript concludes by suggesting image analysis as a practical alternative to spectral analysis, particularly for detecting whether methylene blue has been adsorbed onto the macromolecular network. These findings significantly enhance the understanding of the complex interactions between methylene blue and gelatin in a membrane and lay a solid foundation for future research in this field.

## 1. Introduction

Ultraviolet-visible (UV-VIS) spectroscopy is a well-established technique for studying substances that absorb radiation within the visible spectrum. This method proves particularly useful for examining the release of low molecular weight substances [1,2,3] or the diffusion [4,5,6] of substances across membranes. Its significance lies in its capability to offer a detailed analysis of chemical structures and interactions. However, the use of spectroscopy necessitates complex laboratory equipment, which can potentially hinder its application in miniaturized settings [7,8]. Furthermore, UV-VIS spectroscopy measurements are time consuming, which poses no problem in standard procedures where autosamplers handle mass measurements. Nevertheless, this time factor may limit comprehensive and large-scale analyses, as well as analyses requiring a high frequency of consecutive measurements. Additionally, to obtain local spectrum data, such as that from a point with a radius of 10 µm, it becomes necessary to acquire a specialized UV-VIS imaging spectroscopy device [9].

This study introduces image analysis as an economical, fast, and relatively precise alternative [10,11]. This approach provides a way to streamline and expedite the analytical process, potentially enhancing efficiency and scalability in both research and industrial applications. However, this method has its limitations. The range of colors it can analyze is significantly limited [12,13]. The substance being studied must exhibit a distinguishable response, separate from other factors that might complicate the primary effect. This article demonstrates the application of image analysis, using the example of gelatin and methylene blue, which, in our assessment, is a suitable case for the use of this method.

Layered hydrogels are composite materials comprising hydrogel layers stacked sequentially [14]. These materials encompass unique properties and customizable functionalities for a wide range of applications [15]. Several techniques are commonly used to create these layered hydrogels. The simplest method involves physically stacking individually prepared hydrogel layers [16], achieved using techniques like spin-coating, dip-coating, or casting and subsequently assembling them to form a multilayer structure.

The layer-by-layer assembly technique [17] involves the step-by-step deposition of alternating layers of hydrogels with opposite charges. For instance, one layer could be composed of a negatively charged polymer, such as alginate or hyaluronic acid, while the next layer might consist of a positively charged material, like chitosan or poly(ethyleneimine). Polyelectrolytes typically act as the fundamental components. These layers are created via electrostatic interactions between the charged elements, allowing for precise control over the thickness and composition of each layer.

Covalent bonding offers another method for the creation of layered hydrogels [18]. This process involves the chemical cross-linking of individual hydrogel layers by introducing reactive groups, such as amines or thiols, onto the surfaces of the hydrogels, followed by initiating cross-linking reactions between the layers. Photolithography techniques, commonly employed in microfabrication, can be adapted for producing patterned or layered hydrogels [19]. Light-sensitive hydrogel precursors are exposed to specific light patterns via a photomask, resulting in selective cross-linking and the formation of desired layers. Microfluidic devices provide an additional approach for generating layered hydrogels with precise architectural control. Various hydrogel precursors are introduced into separate channels within the microfluidic device, and the controlled mixing or layering of these fluids leads to the creation of layered hydrogels. Three-dimensional printing [20] and additive manufacturing techniques can be applied to fabricate layered hydrogels with intricate geometries. Specialized 3D printers capable of handling hydrogel materials can deposit multiple layers in a controlled manner, facilitating the creation of complex structures.

The concept of multilayered membrane diffusion is well documented in the literature, with numerous theoretical studies providing an in-depth understanding of molecular transport across different membrane layers. However, real-world systems and their diffusion processes often prove to be more complex than theoretical models might suggest.

A significant portion of experimental research in the field of diffusion primarily centers on the process of diffusion via cellular membranes. The transport through cell membranes is a complex issue, as described in the review by Flemming et al. [21]. Multilayered diffusion is a concern, as it deviates from the simple diffusion model, and its mechanism is more precisely defined at the molecular level. Simple diffusion typically pertains to the transport of small molecules, such as gases [22] or fatty acids [23]. This tri-layered structure contributes to the unique selectivity and permeability characteristics of the cell membrane. Furthermore, numerous studies investigate diffusion across artificially created tri-layered structures, with many drawing parallels to natural cellular membranes.

At its core, a cell membrane comprises artificially synthesized three-layer membranes designed to mimic specific functions in electrochemistry [24] or water purification processes [25]. For example, Ling et al. explored the self-assembly design of multilayer materials using hydroxyapatite and protein nanofibrils, resulting in a structure resembling the layered composition of a seashell [26]. While they studied the permeation of various compounds via this layered structure, they did not investigate the concentration within the membrane itself.

In our article, we shed light on a similar multilayered structure in which hydrophobic layers are created from polypropylene textiles while the hydrophilic components are derived from gelatin. Our objective is to ascertain the concentration within the membrane.

The next aspect of this article introduces the idea of substituting spectroscopic analysis with an RGB model [27,28,29]. The utilization of RGB indices is not widely employed, primarily due to its relatively lower precision. Typically, it is used for quick or preliminary analyses, such as in digital image processing and color analysis across various fields, but not as a direct replacement for UV-VIS spectroscopy [30]. The RGB color model has found practical applications where color information extracted from an image can be harnessed for quantitative or qualitative analysis. For example, in agricultural sciences [31,32], RGB indices have been employed to assess plant health [33] and monitor crops [34]. Some researchers are exploring the integration of image analysis [35], machine learning [36], and computer vision [37] techniques into traditional spectroscopy to expedite the process and reduce costs. In this context, RGB indices could serve as a faster and more cost-effective alternative to UV-VIS spectroscopy.

**Figure 1:**
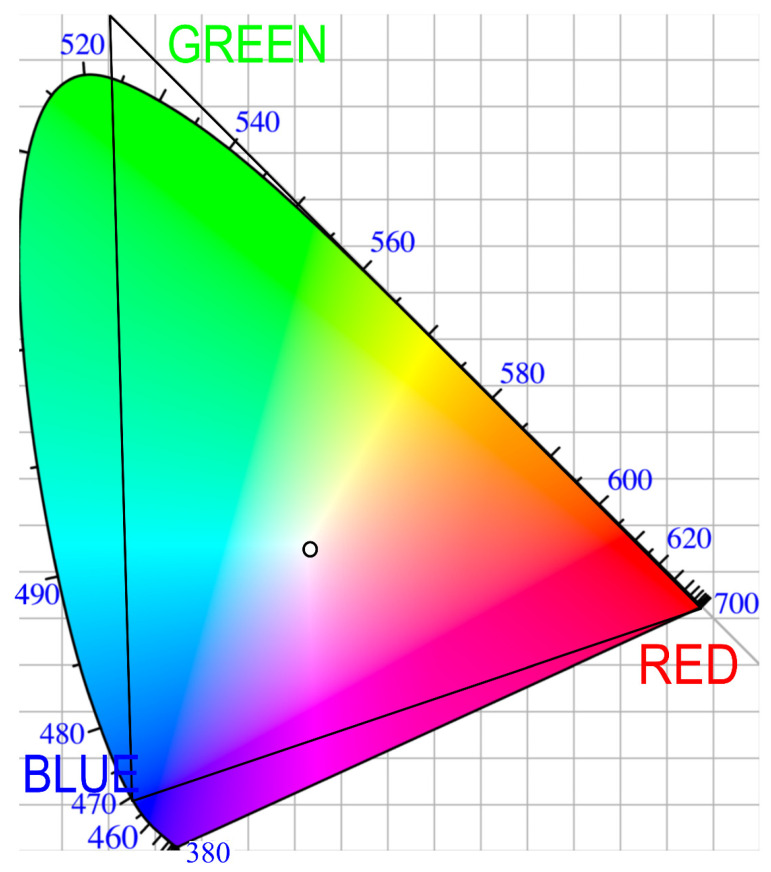
The CIE (Commission Internationale de l’Eclairage, 1931, [38]) chromaticity diagram; the RGB space, denoted by the triangular area; the colors that fall outside the RGB triangle cannot be combined from RGB, underscoring the limitations of this color representation system.

The fundamental principle and the extent of color representation simplification can be illustrated using the CIE (Commission Internationale de l’Eclairage, 1931, [38]) chromaticity diagram, as shown in Figure 1. This diagram is centered around point E, which symbolizes white or transparent color. The angular axis around this central point represents hue (wavelength), while the radial axis signifies intensity. These angular and radial axes are then transformed into xy coordinates, with the resulting coordinates specifying a particular color shade.

A plausible combination of wavelengths for color representation could include 630 nm for red, 532 nm for green, and 465 nm for blue light, often referred to as RGB. This RGB system maps out a triangle within the color space, resembling the process of converting analog images into digital format. Nevertheless, it is important to recognize that such simplification comes with inherent limitations.

As shown in the diagram, certain colors fall outside the boundaries of the RGB triangle and thus cannot be accurately represented within this system. Additionally, some values within the RGB space may not precisely correspond to their true colors. For example, cyan light, with a wavelength of 490 nm, does not perfectly align with its exact hue in the RGB representation and requires approximation to the nearest color within the RGB triangle.

The literature has explored the substitution of visual light spectra using the RGB method. Notably, a study conducted by Xiao et al. [39] focused on observing the iodine–starch system. In this system, starch undergoes a significant color shift from red to blue, as seen in the well-known iodine–starch test. Another study examined the reaction of the tetraphenyl cyclopenta-derived Schiff base with cyanide ions, which was employed for the quantitative analysis of CN ions in solution [40]. This method can be applied to numerous systems. In our own research, we introduced a method to study the interaction between methylene blue and gelatin. Our technique offers the additional advantage of image analysis, which enables the scanning of individual pixels. The appropriate light models can be applied in fluorescence studies [41].

While the RGB model is widely used, alternative models such as CIELAB are also available. CIELAB is currently the most commonly used system for quantitatively describing the color of an object. This preference is due to its uniformity, ease of acquisition, low cost, and device independence [42]. The CIELAB model is grounded in physical interpretations and consists of three parameters: l* (lightness), a*, and b*. The a* and b* parameters correspond to the four unique colors in human vision, red, green, blue, and yellow, arranged in a 2D coordinate system. Specifically, the a* parameter is located on the red–green axis, while the b* parameter is positioned on the blue–yellow axis.

Another relevant model is the CIE HSL, characterized by three parameters: hue, saturation (or chromaticity), and lightness. In this system, hue corresponds to the wavelength, saturation denotes the intensity of light, and lightness scales between white and black. To date, no direct method exists for converting spectra to the CIE LAB or HSL color space. Current practices involve first transforming spectra into the CIE chromaticity diagram (as shown in Figure 1). Subsequently, the diagram can be converted into the CIE LAB [43] or CIE HSL model [44]. A direct transformation of spectra into the CIE models has not been identified.

## 2. Results

This paper provides an account of a series of tests conducted on samples to investigate diffusion processes, with a specific focus on the diffusion of methylene blue across a layered membrane constructed from polypropylene nonwoven textiles. Hydrogel is utilized as a substance capable of binding and releasing substances. Notably, we have observed that a simplified RGB model, rather than spectroscopy, can serve as an effective analytical tool.

**Figure 2:**
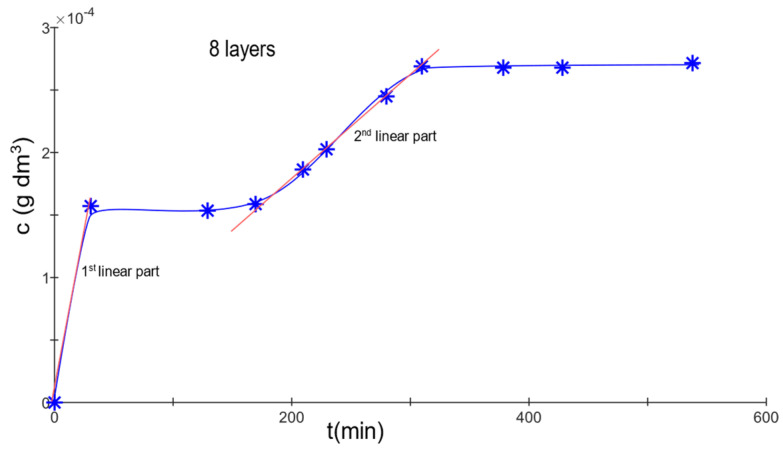
Time evolution of methylene blue concentration released from an 8-layered material. The graph represents the dynamics of dye release over time. While not shown here, similar patterns of release were observed for 4-, 16-, and 32-layered materials at different time scales.

Model materials, consisting of 2, 4, 8, 16, and 32 layers interconnected with gelatin hydrogel, were created. A diffusion test with methylene blue was performed on each sample, monitoring the transition of a specific dye across the membrane. The diffusion process was interrupted upon the detection of a second wave of diffusion (see Figure 2). These two waves of diffusion represent distinct processes. The first wave signifies the migration of dye that was not bound to the hydrogel across the membrane. This dye circulates freely in the solution, with its movement solely influenced by the membrane itself.

The second wave indicates the transition of dye that was partially bound to the hydrogel as it moves through the membrane. This suggests that during the initial phase of diffusion, the hydrogel absorbed a portion of the dye, which it is now releasing back into the solution, allowing it to diffuse across the membrane. Consequently, this second wave is triggered by the release of dye from the hydrogel.

By monitoring the concentration of solutions in both cells at different time intervals, it is possible to establish a relationship that can be used to calculate the diffusion coefficient [45].

(1) $$D_{eff}=\left(\frac{dn}{dt}\right)\left(\frac{l}{\Delta c}\right)$$

In this equation, the following are used:

*D_eff_* represents the effective diffusion coefficient of the solute in the solvent.

(*dn*/*dt*) is the concentration gradient.

*l* is the membrane’s thickness.

∆*c* is the difference in concentration between the source and receiving cells.

The diffusion coefficients were determined for the initial wave of diffusion. Based on the measurements of the effective diffusion coefficient, it became evident that for the two-layered membrane, diffusion occurred rapidly (*D_eff_* = 1.2 × 10^−9^ m^2^·s^−1^). However, upon recalculating for a single layer, the coefficient was noticeably smaller. Given that the material consisted only of a thin gelatin layer, the dye had a short path, suggesting a direct route. This allowed for the observation of methylene blue’s passage within the first minute. In contrast, the four-layered membrane, which used a greater quantity of polymer to bind the nonwoven fabric, presented a different scenario. The hydrogel likely filled the nonwoven fabric’s pores (about 20 µm), creating more complex pathways for dye diffusion. Still, with these materials, there was a notable absence of significant retention of methylene blue, possibly due to the membrane’s relative thinness, which allowed the solvent (water) to permeate more easily. For membranes comprising 8, 16, and 32 layers, diffusion rates remained relatively consistent, with *D_eff_* = 25 × 10^−9^ − 28 × 10^−9^ m^2^·s^−1^ when the diffusion coefficient was recalculated for a single layer.

The secondary diffusion wave was modeled using a linear function, correlating concentration over time. The average effective diffusion coefficients for 2 and 4 layers were immeasurable. However, for 8 layers, the coefficient was 15.3 × 10^−9^ m^2^·s^−1^; for 16 layers, it was 3.5 × 10^−9^ m^2^·s^−1^; and for 32 layers, the value stood at 1.32 × 10^−9^ m^2^·s^−1^.

Upon the conclusion of the second wave of diffusion, the experiment progressed to the next phase. The membrane was carefully removed from the solution and thoroughly dried. The drying step was essential for subsequent analysis, as a dried membrane offers improved stability and ease of handling. During the drying process, all moisture evaporated from the membrane, preventing any further dispersion of water within the membrane. It was crucial to handle the membrane with care to avoid any damage that could distort the results.

**Figure 3:**
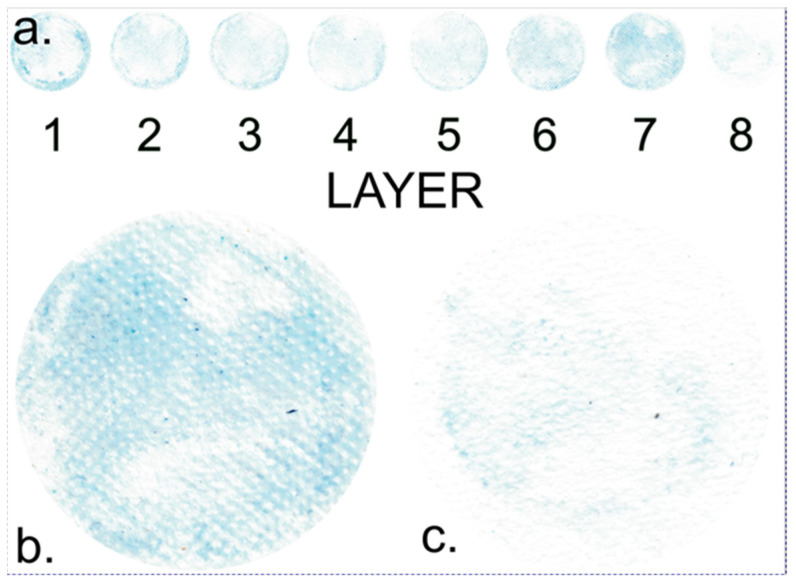
Layer-specific scans of an 8-layer sample. (**a**) Scans representing all layers. (**b**) Scan of the most dye-intensive layer (7/8). (**c**) Scan of the least dye-intensive layer (8/8). This figure provides a comparison of dye distribution across different layers of the sample.

The subsequent phase involved dissecting the membrane into individual layers, facilitating a detailed analysis of each layer separately. A membrane comprising multiple layers can influence diffusion processes in various ways. Subsequently, the concentration of the dye was determined in each individual layer. This analysis provides detailed insights into how the dye dispersed through the membrane during the diffusion processes. For example, determining the dye concentration in individual layers can reveal whether certain layers allow the passage of dye or if some layers absorb more dye. This method offers a comprehensive understanding of how different layers of the membrane affect substance diffusion. In this model, all layers share the same composition, each constructed from polypropylene nonwoven fabric with identical porosity. Figure 3 provides a detailed scan of individual membrane layers following the diffusion process. This scan was conducted to visualize and quantify the distribution of the dye across different layers of the membrane.

Upon examining the image, it is evident that the highest concentration of dye is found in the outer layers of the membrane, which are in direct contact with the solution. This observation aligns with expectations since the dye diffuses into the membrane from the solution, and the outer layers are the initial points of contact. As a result, these peripheral layers, due to their proximity to the solution, tend to retain most of the dye. Interestingly, an increased concentration of blue dye is also observed on the opposite side of the membrane, which is adjacent to the less concentrated solution. This phenomenon will be discussed later.

In contrast, the internal layers of the membrane exhibit lower dye concentrations, suggesting that the dye may require some time to permeate the full depth of the membrane. This finding is consistent across all other samples.

Figure 3b,c provide detailed scans of individual membrane layers following the dye diffusion process. These visual representations reveal patterns that offer further insights into the distribution of the dye within the membrane. The figures indicate that the dye distribution is not uniform. Existing research suggests that the dye is strongly adsorbed onto the gelatin macromolecules, significantly influencing its movement and distribution across individual layers.

**Figure 4:**
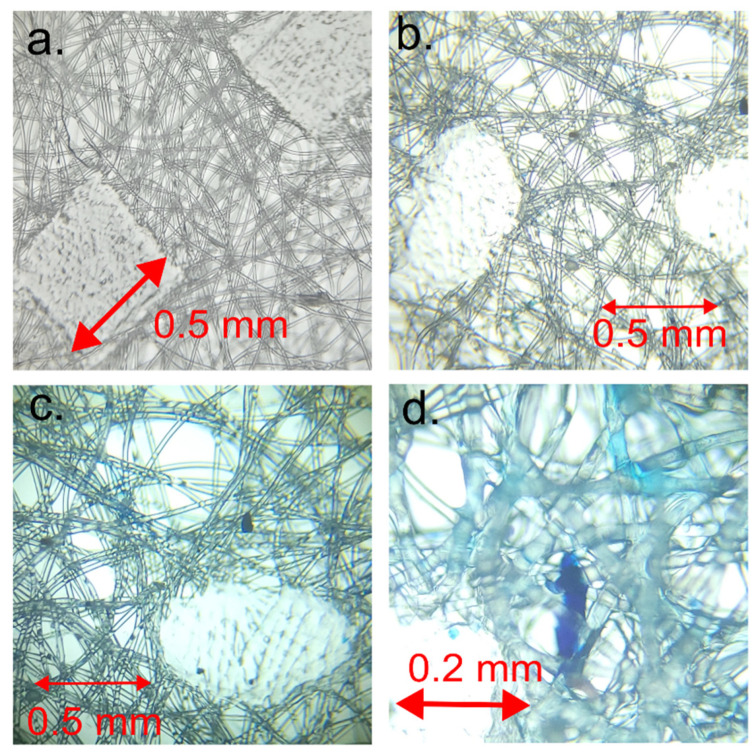
Microscopic image of material: (**a**) new, unused nonwoven textile; (**b**) layer with low intensity of blue color (the macroscopic appearance of the textile is white); (**c**) layer with high intensity of blue color (layer also has visually blue appearance); (**d**) zoomed sample from Figure 4c.

Notably, the images show square domains within the layers, devoid of dye adsorption, which stand out from the rest of the membrane. These square domains result from the melting of textile material at these points during production (Figure 4a), causing the polymer in this domain to lose its fibrous structure. Consequently, these square domains absorb minimal dye, appearing lighter. It can be inferred that the area that lost its fibrous structure will bind a smaller quantity of gelatin.

Nonetheless, these melted square domains play a crucial role in maintaining the overall structure of the membrane. Their presence reinforces the membrane and helps maintain its stability throughout the diffusion process. Without these polymer domains, the membrane could be more susceptible to deformation or damage during the process.

Figure 4a provides a visual representation of the microstructure of the square domains. This image was obtained via the microscopic imaging of a single membrane layer and offers detailed information about the distribution of these domains within the membrane. These square domains, composed of melted polymers, are easily identifiable due to their low dye absorption.

This comprehensive examination of the membrane and its composition not only enhances our understanding of dye distribution within the membrane but also sheds light on how various membrane components influence this process. Based on these images and other data, hypotheses can be formulated regarding the impact of these domains on the diffusion and stability of the membrane and how its composition can be modified to better suit specific requirements.

Microscopic imaging reveals that the areas between the polymeric square domains consist of randomly oriented fibers. These fibers, distinctly blue, absorbed the dye used in the experiment. It is conceivable that these randomly oriented fibers are modified by gelatin. Unlike the molten domains, which absorb minimal dye, these fibrous areas absorb the dye more extensively, resulting in their blue appearance.

The adsorption process is influenced by various factors, including the material’s chemical structure, porosity, and affinity for the adsorbed substance. In this case, the fibrous areas of the membrane exhibit a strong affinity for the dye and effectively adsorb it. It is also possible that the random orientation of the fibers contributes to the adsorption and distribution of the dye within the membrane. The random orientation of the fibers may create more space, facilitating the diffusion of the dye.

The spaces between the melted square domains are occupied by a structure comprising gelatin (Figure 4b). This is where the methylene blue is found within the gelatin. Gelatin is a polymer capable of forming a gel-like structure in aqueous solutions. Since it is transparent, it is not visible in the spaces between the fibers and can be observed as a shadow surrounding the fibers. Grains of gelatin with dye can be discerned, appearing darker than other elements. It is assumed that the light blue opalescence of the fibers arises from the methylene blue, which precipitates but does not adsorb onto the surface of the fibers during the drying process. This precipitation occurs during the sample’s drying phase.

These gelatin structures are visibly colored blue, a result of the blue dye’s adsorption. The experiment established that gelatin exhibits a pronounced affinity for this dye and the capacity to effectively adsorb it.

The membrane structure comprises two primary components: the square domains consisting of melted polymers, which absorb a minimal quantity of dye and provide structural reinforcement and stability to the membrane, and the gelatin-filled regions between them. These gelatin regions function as channels for dye diffusion and effectively adsorb it.

**Figure 5:**
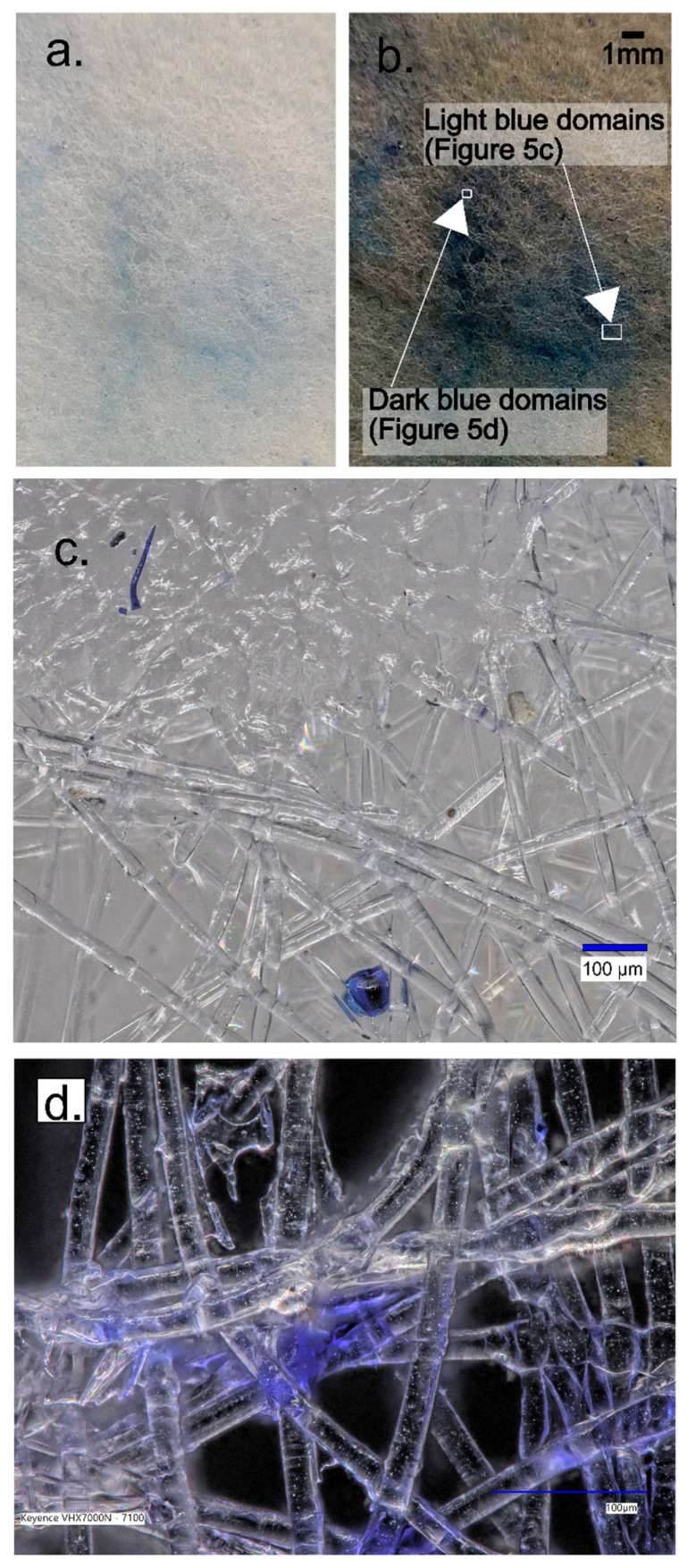
High-resolution photography of the sample highlighting two types of blue pixels and the presence of white square domains. (**a**) Original photography; (**b**) the same snapshot with increased contrast. This high-resolution image provides further insight into the color variation and the spatial distribution within the sample; (**c**) zoom of light blue domains provided by 4K high-resolution microscope; (**d**) zoom of dark domains from 4K high-resolution microscope.

Detailed imagery reveals the presence of pixels in two distinct shades (Figure 5), potentially indicating different states or properties of the examined material, which, in this case, is the membrane.

The first shade could represent regions where dye adsorption is significant. These regions are likely to correspond to the gelatin structures located between the square domains, as discussed earlier. Given gelatin’s propensity to adsorb dye, these regions might exhibit more intense coloration. Conversely, the second shade could denote areas with a lower dye concentration.

Therefore, the varying shades might reflect differing degrees of dye adsorption within the membrane. This in-depth examination of the membrane enhances our understanding of its structure and function, enabling the identification of regions with varying adsorption capabilities.

The high-resolution 4K digital microscope, Keyence VHX-7000, allowed us to magnify and examine the light and dark domains. In Figure 5c, the light domains were characterized by the presence of blue-colored grains distributed within the textile fiber structure. Conversely, the dark domains were a result of hydrogel adsorption, with the opalescent color dispersion occurring within the hydrogel matrix.

**Figure 6:**
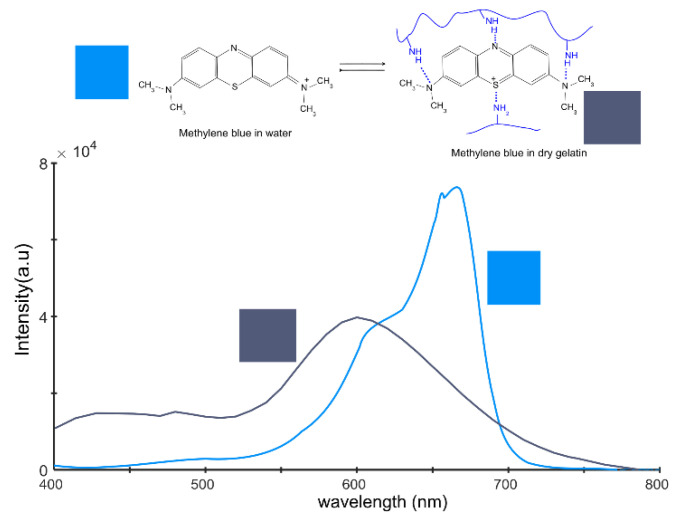
Two spectra for methylene blue, representing resonance structures 1 and 2. These spectra provide key insights into the spectral behavior of methylene blue under different conditions, underscoring its versatility as a probe in various experimental setups.

Methylene blue, an organic dye, is renowned for its unique properties, which include its ability to exist in two distinct resonance states (Figure 6). Resonance, a concept derived from quantum mechanics, pertains to a molecule’s capacity to exist in multiple energetically equivalent structures. In the case of methylene blue, these states involve varying molecular conformations, each associated with unique light absorption properties.

The first resonance state of methylene blue predominates when the dye is solvated in water or in a desiccated, crystalline state. This state is characterized by a specific light absorption spectrum, which can be observed via spectroscopy.

The second resonance state typically emerges when methylene blue interacts with another potent molecule via hydrogen bonding [46,47,48,49]. This interaction alters the energy state of the methylene blue molecule, resulting in the second resonance state. This state presents a distinct absorption spectrum, leading to a noticeable change in how methylene blue absorbs light.

The transition between these two resonance states forms the basis for many applications of methylene blue, including its use as an indicator in chemical and biochemical experiments. Methylene blue’s ability to modify its absorption spectrum in response to interactions with other molecules is advantageous for monitoring these interactions and gaining insights into molecular behavior.

To perform the spectrum conversion, we utilized a script titled “Convert Spectrum to Color,” developed by Tingbiao Guo [50]. For methylene blue freely solvated in water, the RGB conversion results in RGB = [0, 0.57, 0.97]. In the standard 8-bit depth format with 256 intensity levels, this corresponds to the color [0, 145, 248]. In the case of the second resonance structure, the RGB ratio is RGB = [0.32, 0.36, 0.47] or [81, 91, 121] in the 8-bit depth format.

Alternatively, the spectra were recalculated into the CIE-LAB parameters, where the first color has the parameters [l = 59, a = 5.6, b = −60], and the dark blue spectra have parameters [l = 39, a = 6, b = −17]. The CIE LAB parameters are relatively abstract. The only parameter connected to a physical property is the l* parameter, which measures lightness. This is why photographers often use the CIE-HSL models, where l represents lightness, s denotes saturation or chroma, and hue signifies the shade of the material. In this approach, the first spectra have parameters [l = 59, c = 60, h = 275], and the second spectra have parameters [l = 39, c = 18, h = 281].

Both the CIE LAB and CIE HSL models are more related to human perception of colors. In contrast, the RGB model is based on the physical mixing of colors. The lightness in both models is perceived on a scale from 0 to 100, where 0 represents extremely dark, approaching black in perception, and 100 represents extremely light, approaching white.

The range of hue parameters is from 0 to 359, which corresponds to the angle in a color wheel. An angle of 0 represents the red color with a wavelength of 700 nm, and an angle of 359 represents the violet color with a wavelength of 400 nm. The parameters between these limits cover the range of all spectra. Saturation falls within the range of 0–100 and indicates whether a color is perceived as gray or colored.

In an ideal scenario, a spectroscopic analysis of each individual pixel on the membrane would be conducted to accurately characterize the spectral response of methylene blue in different areas. This would provide direct information about the presence and distribution of both resonance states of the dye. However, this procedure is technically challenging and often impractical, particularly when numerous pixels require analysis.

Moreover, conducting a spectroscopic analysis of the entire surface simultaneously would yield a mixed spectrum of both resonance structures of methylene blue. Interpreting this spectrum would pose challenges, as the absorption bands of both states might overlap, thereby complicating the interpretation of the results.

However, an alternative approach can help circumvent these challenges. It is possible to exploit the fact that the light spectrum reflected off a material manifests in the visible spectrum as a combination of three primary colors: red, green, and blue (RGB). These colors form the basis for all other perceivable colors, and their ratio and intensity dictate the shade and brightness of the observed color.

RGB (red, green, and blue) analysis represents a simplified form of spectroscopic analysis. Although it might not capture the spectrum’s subtle nuances as spectroscopy does, it serves as an effective method for characterizing materials’ color properties.

The RGB model operates on the additive color mixing principle, wherein the three primary colors—red, green, and blue—can be variously combined in different ratios and intensities to create a wide array of shades. Each of the three-color components can assume values from 0 (no light of this color) to 255 (maximum light intensity of this color), leading to a total of 2 to the power of 24 possible color combinations, or approximately 16.7 million shades. While finite compared to the continuous spectrum of potential wavelengths in spectroscopy, this figure still offers a vast assortment of color shades for analysis.

**Figure 7:**
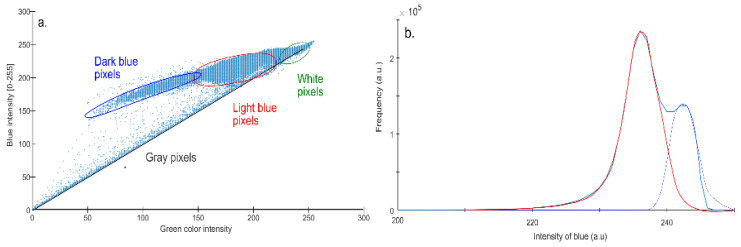
(**a**) Two-dimensional red–blue histogram of all pixels in the scan. This provides visualization of the overall distribution of pixel intensities. (**b**) 1D histogram of blue pixels only (white, transparent, gray, and black pixels were excluded). The histogram offers a focused view of the distribution of intensities, specifically within the blue range.

RGB analysis permits the organization of color shades into a three-dimensional matrix, facilitating data visualization and analysis. An image illustrating the distribution of blue and green values in a two-dimensional graph represents this feature (Figure 7a).

According to this distribution, several groups of colors are identifiable. The first group, situated along the graph’s diagonal, encompasses various gray shades. These gray shades likely represent artifacts generated during the scanning process. Considered noise, they were eliminated before further analysis to minimize their potential disruptive influence.

The subsequent group is represented by a cloud of points located in the blue shade range between 180 and 210. This region contains the first shade of blue, likely corresponding to one of methylene blue’s resonance states.

Another cluster of points, this time with a higher intensity of around 240, represents a different shade of blue. This shade might correspond to methylene blue’s second resonance state.

It is vital to understand that the image’s color intensity does not directly correspond to the spectrum’s intensity. The color intensity can fluctuate depending on the given substance’s concentration. However, it is plausible to assume that the intensity distribution of a specific color around its average value should adhere to a Gaussian (normal) distribution.

In this context, we conducted an analysis of pixels exhibiting blue color. The blue shades’ values of these pixels were collated into a histogram (Figure 7b). This histogram revealed two distinct peaks corresponding to two different blue shades, suggesting the presence of two distinct methylene blue resonance states.

Subsequently, we executed a deconvolution of these peaks and determined the total number of pixels corresponding to each blue shade. This procedure facilitated a representation of individual shades on the membrane surface.

**Figure 8:**
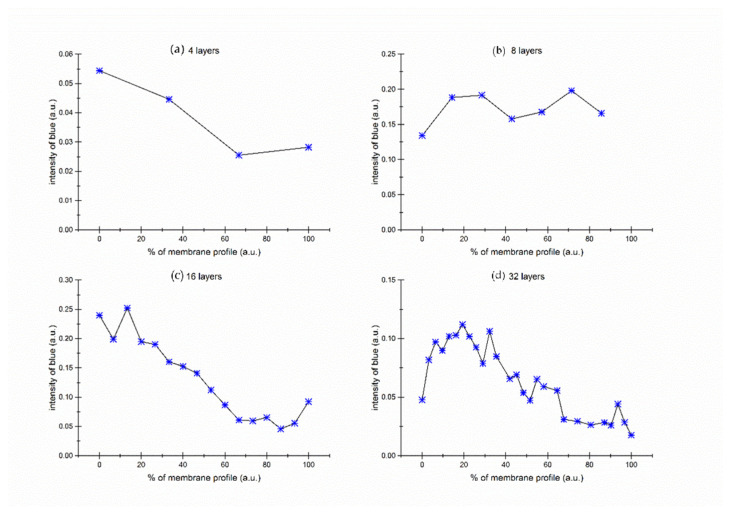
Concentration profiles of the blue dye not bound to gelatin across the layer thickness—fraction of blue pixels from all pixels in the layer: (**a**) 4 layers; (**b**) 8 layers; (**c**) 16 layers; (**d**) 32 layers; dashed lines represent the diffusion interface.

Figure 8 illustrates the area concentration of the blue dye on the membrane, calculated as the ratio of blue pixels to the total number of pixels in the scanned membrane area.

Two significant effects are observable within this image. The first effect (Figure 8a,b) is evident in thin membranes, specifically those consisting of 2, 4, or 8 layers. In these instances, the concentration of methylene blue remains constant across the membrane. This constancy is likely attributable to the diffusion process, during which the dye permeates the membrane, leading to an equilibrated concentration across all layers. As a result, the dye distributes uniformly across the membrane. The experiment with 8 layers membrane was repeated 3 times. The result of repeated analysis is presented in Supplementary Material (Figure S1).

Conversely, in thicker membranes—particularly those with 16 or 32 layers—a diffusion interface forms. This interface denotes an area where a gradual shift in dye concentration occurs between both membrane edges. The edge proximal to the source solution exhibits a higher dye concentration, as it serves as the initial point of dye adsorption. On the opposite edge, closer to the target solution, the dye concentration is lower. Here, the dye has predominantly diffused and is also partially adsorbed into the membrane’s inner layers.

Within the membrane’s inner layers, the dye concentration varies according to a sigmoid function, representative of the diffusion concentration gradient. This signifies a gradual decrease in dye concentration from the edge adjacent to the source solution toward the edge near the target solution, demonstrating the dye’s progressive diffusion across the membrane layers. This gradual process results in the formation of a diffusion concentration gradient, discernible from the contrasting dye concentrations at both membrane edges and the gradual concentration shift within the inner layers.

**Figure 9:**
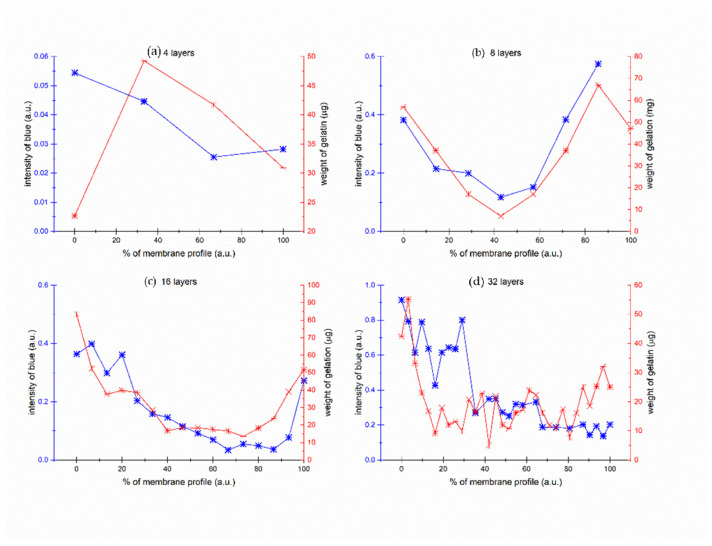
Concentration profiles of blue dye bound to gelatin across the thickness of various layers. The red right axis represents the weight of gelatin adsorbed on the surface of the nonwoven textile—a ratio of blue pixels to all pixels within each layer. The different sub-figures represent (**a**) 4 layers; (**b**) 8 layers; (**c**) 16 layers; and (**d**) 32 layers, respectively.

Figure 9 illustrates the correlation between the concentration of blue dye, hypothesized to be bound to gelatin, and the weight of gelatin per unit surface area of the nonwoven fabric. To facilitate a clear comparison of these dependencies, both primary and secondary axes are aligned at zero. This alignment enables a clear observation of the proportionality between these two functions.

The graph reveals an apparent correlation between the presented data sets, suggesting that fluctuations in the concentration of blue dye are linked with alterations in the weight of gelatin per unit surface area of the nonwoven fabric. This correlation emphasizes the crucial role that gelatin plays in the dye’s diffusion and adsorption processes within these membranes. The experiment from Figure 9b with 8 layers membrane was repeated 3 times. The result of repeated analysis is presented in Supplementary Material.

**Figure 10:**
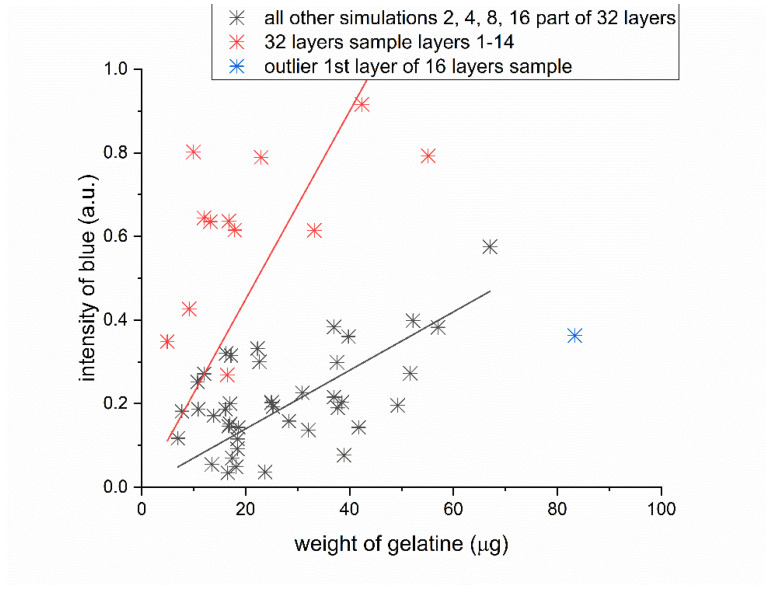
Data from all four previous scenarios offering a comprehensive view of the relationship between the weight of gelatin adsorbed onto the nonwoven fabric and the quantity of dye bound to this gelatin. This consolidated visualization enhances our comprehension of the dynamics of these interactions. It also enables us to discern patterns and trends in these processes that could potentially be missed when examining the data in a segmented manner.

Figure 10 consolidates data derived from the four previous scenarios depicted in Figure 9. It outlines the relationship between the weight of gelatin absorbed by the nonwoven fabric and the quantity of dye to which the gelatin adheres. This collective visual representation enhances our understanding of the dynamics involved in these interactions. Moreover, it enables us to identify patterns and trends that may be potentially overlooked when examining the data in isolated segments.

The paired data—gelatin weight and blue intensity—for all samples broadly segregate into two clusters. Pairs from the 2-, 4-, 8-, 16-, and 32-layer sets predominantly aggregate within one cluster. An exception is evident for selected samples from the 32-layer set, specifically layers 1–14, indicated as red points. These samples, which are the thickest ones (32 layers), exhibit a more intense blue hue when in contact with the source solution, denoting a higher dye concentration and implying enhanced adsorption.

In the case of the 32-layer samples, there are instances where the gelatin permeates the nonwoven fabric so thoroughly that it also envelops the polypropylene squares from the melted polypropylene, which appear white in other samples.

## 3. Discussion

Two critical points are discussed in this study: the potential substitution of UV-VIS spectroscopy by the RGB model and the intriguing distribution of gelatin and methylene blue.

The substitution of UV-VIS spectroscopy with the RGB model offers several advantages, including higher measurement frequency, simplicity, and the ability to utilize common and affordable devices like cell phones or office scanners for precise analysis. Several examples of this application can be found in the literature, often in simplified versions where changes in liquid color indicate concentration changes. This study takes the RGB model further by incorporating image analysis, allowing the differentiation of two different samples from a single scan. Image analysis is a valuable tool for assessing dye diffusion and absorption, especially in cases where traditional spectroscopic analysis may not be practical or suitable. However, it is important to consider the limitations of this method, as it may not always replace spectroscopic analysis, particularly when precise substance concentrations are required. The application of this method can be considered from various perspectives.

Image analysis represents a digitalized approach to analog signal interpretation, where the UV-VIS spectrum serves as the analog signal, and the RGB model serves as its digital counterpart. Digitization results in some loss of information, which may not be perceivable by human senses but is still detectable in mathematical models. However, the loss of color information in the RGB model, compared to the continuous UV-VIS spectrum, is relatively minor. While the UV-VIS spectrum can represent an infinite number of color shades, the RGB model is limited to approximately 16.7 million colors. The most significant information loss occurs in intensity. The RGB model does not provide information about the dye’s concentration but rather determines whether the dye is present or absent in each pixel. In contrast, traditional UV-VIS spectroscopy provides the average spectral intensity of all pixels in a sample. Additionally, there is a clear relationship between spectral intensity and concentration in spectroscopy, whereas the RGB model lacks such a direct correspondence. It can only identify the presence or absence of dye on a binary basis.

Traditional spectroscopy methods require the dye to be dissolved in a solution, which may erase information about the dye bound to gelatin, as the resonant structure is only observed when the dye is bound to dry gelatin. Most UV-VIS spectrometry techniques rely on transparent samples, making it impossible to measure the spectrum of such materials. This limitation underscores the advantage of image analysis, which can often measure only reflected light.

The next focus of this study is on the investigation of layered materials. There are limited articles in the literature that explore diffusion via multilayer materials, and little information is available on the distribution of color in the membrane. Although some studies have presented diffusion profiles in multilayer materials and their potential use in water purification, specific concentration profiles within the membrane are lacking. It is believed that the concentration inside the membrane will follow a sigmoidal pattern. However, the distribution of methylene blue and gelatin in the membrane’s profile was rather unexpected. Initially, it was hypothesized that the dye’s distribution in the membrane would resemble a standard diffusion interface. While there has not been any statistical evaluation, the trends are consistent across all samples, and one sample was analyzed three times, indicating a systematic property.

Many questions remain unanswered, offering opportunities for further investigation. Is this behavior specific only to gelatin and methylene blue, or does it extend to other substances? The concentration profile represents a snapshot of the situation, and understanding the timeline of this process could provide insight into the diffusion mechanism. The experiment began with a dry membrane, but would the results be the same if the experiment started with swollen gelatin instead of a dry membrane? The wide range of experimental activities could lead to the development of new types of membranes better suited to specific requirements.

This study initially hypothesized that the highest concentration of dye would be in the layer adjacent to the source solution, with a lower concentration in the layer adjacent to the target solution, resulting in a diffusion interface with a concentration gradient. However, this hypothesis was not supported by the findings. In all samples, the edge parts exhibited the highest concentration, while the concentration was lowest in the middle.

When considering only the dye that is not bound to gelatin, whose movement is regulated by diffusion in water (although slowed by the presence of a macromolecular network and nonwoven textiles), this initial assumption is validated, and a diffusion interface is observed. Conversely, a correlation was discovered between the gelatin and the second type of dye, suggesting that the dye bound to the gelatin moves in concert with the gelatin.

The observed higher concentration of gelatin in the edge layers compared to the middle is attributed to the initial dry state of the sample when inserted into the apparatus. During the initial phase, a swelling gradient was established from both sides, with the periphery of the sample in contact with the solution first, becoming more concentrated. The dye bound to the gelatin moved along with this gelatin movement.

## 4. Conclusions

This paper delves into several facets of methylene blue dye, including its behaviors, interactions with gelatin in a membrane environment, and the application of analytical methods such as spectroscopy and image analysis. The literature has documented the existence of two resonance states of methylene blue, each exhibiting distinct light absorption properties within the visible spectrum. These states can be distinguished using the RGB model.

This study employs image analysis as a viable alternative to spectral analysis, particularly in the detection of methylene blue adsorption onto the macromolecular network. This innovative approach has proven to be valuable in assessing the interactions between the dye and the membrane structure. The research findings reveal significant color diffusion concurrent with gelatin migration, indicating a strong binding affinity between methylene blue dye and gelatin.

Furthermore, this study offers insights into the interplay between concentration gradients and diffusion processes within the membrane. The unexpected concentration profiles of the dye and gelatin across the membrane, with the highest concentration at the periphery and the lowest in the center, shed light on the complex dynamics of substance migration in such systems.

Additionally, the study investigates the hydrophilic properties of gelatin and its impact on dye diffusion in the presence of a concentration gradient. The findings suggest that when the dye is bound to gelatin, it tends to migrate along with the gelatin, particularly toward regions of higher water concentration.

In conclusion, the insights derived from this discussion significantly enhance our understanding of the intricate interactions between methylene blue and gelatin within a membrane environment. These results provide a strong foundation for future research in this fascinating and important field, with the potential to contribute to advancements in membrane technology and its various applications.

## 5. Materials and Methods

### 5.1. Chemicals and Devices

We used polypropylene nonwoven fabric with a specific weight of 17 g m^−2^ (Boltzmann); Bovine gelatin (50–100 kDa) (Sigma-Aldrich Co., St. Louis, MO, USA), CAS: 9000-70-8; and Methylene blue hydrate (319.86 Da) (Penta s.r.o., Praha, Czech Republic), CAS: 122965-43-9.

We used side-by-side diffusion cells (PermeGear, Hellertown, PA, USA), a U-3900H Spectrophotometer (Hitachi, Tokyo, Japan), and a Scanner Lexmark XM 1140. The scale resolution was 0.1 μg.

### 5.2. Preparation of Membrane

Gelatin (Type B, Mw 50–100 kDa) from bovine skin, obtained from Sigma-Aldrich Co., was employed to create polymer solutions with a 3% (*w*/*w*) concentration. These polymer solutions were subsequently used to assemble layered materials, which were composed of nonwoven fabric pieces measuring 10 × 10 cm.

The layered materials were constructed by connecting individual layers of nonwoven fabric using the gelatin polymer solution. Subsequently, the layered material was left to air-dry at room temperature for 24 h and then cut into circular shapes with a 7 cm diameter.

To produce the layered material, a total of 16 layers of polypropylene nonwoven fabric were required, and these layers were interlocked using the prepared 3% (*w*/*w*) gelatin polymer solution. This process effectively bonded the 16 layers of nonwoven fabric together, resulting in a cohesive layered material.

### 5.3. The Diffusion in Cell

**Figure 11:**
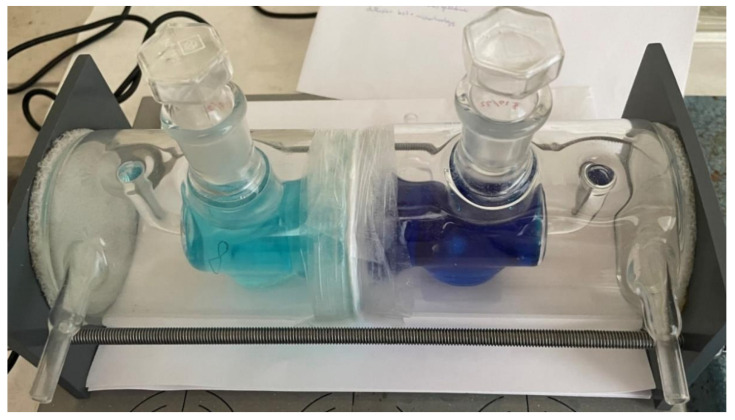
Measurement of the diffusion coefficient on side-by-side cells with a membrane consisting of 8 layers of nonwoven fabric connected by 3% (*w*/*w*) gelatine hydrogel after 8 h.

The materials, prepared as described, were positioned between two diffusion cells and securely fastened within clamps (as illustrated in Figure 11). These diffusion cells were subsequently filled with a solution containing 0.04 g·dm^−3^ of methylene blue in the source cell and distilled water in the receiving cell. The measurements were conducted until the system reached a state of equilibrium. Subsequently, the collected samples were left to air-dry for a minimum period of three months.

### 5.4. Optical Microscopy

The samples were imaged by the Levenhuk 320 BASE microscope with 10× magnification of the ocular lens and 4× magnification of the objective. The zoomed image was measured with 10× magnification of the objective. The microscope was equipped with a digital camera with resolution 3 MPx resolution. The photograph was cropped from the center of the captured image because the margins are distorted.

### 5.5. Scanning of Membrane Profile

The layers of the membrane were carefully separated. The layer was scanned, whereas the orientation of the sample was conserved. The neat sample of nonwoven textile without gelatin and methylene blue was scanned and weighted. The scanning was performed on a white background. The result of scanning was a white bitmap. Next, the scanning of the textile with gelatin was performed, which resulted in the white bitmap. The layer with color was scanned with a resolution of 1200 Dpi. The result is a stack of bitmaps with RGB channels, each channel with 8-bit resolution (256 levels of intensity for each color). The scanned image was exported to a bitmap (bmp) file. The files were processed by MATLAB scripts (version 9.13.0.2166757 R2022b, Mathworks, Three Apple Hill Drive Natick, MA, USA).

## Data Availability

The data presented in this study are openly available in the article.

## Supplementary Materials

The following supporting information can be downloaded at https://www.mdpi.com/article/10.3390/gels9110888/s1. Figure S1: Repeated measurement of blue intensity for sample with 8 layers in the concentration profile.
