# Supplementary material for: Explorative Image Analysis of Methylene Blue Interactions with Gelatin in Polypropylene Nonwoven Fabric Membranes: A Potential Future Tool for the Characterization of the Diffusion Process
Source: Gels. 2023 Nov 9;9(11):888. doi: 10.3390/gels9110888 (PMC10671130; doi:10.3390/gels9110888)
Supplement: Supplementary file 1 [file gels-09-00888-s001.zip › gels-2550426-supplementary.pdf]

Explorative Image Analysis of Methylene Blue Interactions with Gelatin in Polypropylene Nonwoven Fabric Membranes: A Potential Future Tool for the Characterization of the Diffusion Process

Jan Zidek [1,*], Anna Sudakova [1,2], Jiri Smilek [2], Duc Anh Nguyen [3], Hung Le Ngoc [3,4] and Le Minh Ha [5]

1 Central European Institute of Technology (CEITEC), Brno University of Technology, Purkynova 123, 612 00 Brno, Czech Republic

2 Faculty of Chemistry, Brno University of Technology, Purkynova 464/118, 612 00 Brno, Czech Republic

3 Center for Research and Technology Transfer (CRETECH), Vietnam Academy of Science and Technology (VAST), 18-Hoang Quoc Viet, Nghia Do, Cau Giay, Hanoi 100000, Vietnam; ngoc10hung@yahoo.com (H.L.N.)

4 Graduate University of Science and Technology (GUST), Vietnam Academy of Science and Technology (VAST), 18-Hoang Quoc Viet, Nghia Do, Cau Giay, Hanoi 100000, Vietnam

5 Institute of Natural Products Chemistry (INPC), Vietnam Academy of Science and Technology (VAST), 18-Hoang Quoc Viet, Nghia Do, Cau Giay, Hanoi 100000, Vietnam; halm2vn@gmail.com

* Correspondence: jan.zidek@ceitec.vutbr.cz

Supplementary material

Repeated measurement of the intensity of blue for three samples (Figure 8b and 9b from main text), which were prepared with the same method, diffusion was performed for similar time, and they were decomposed to layers.

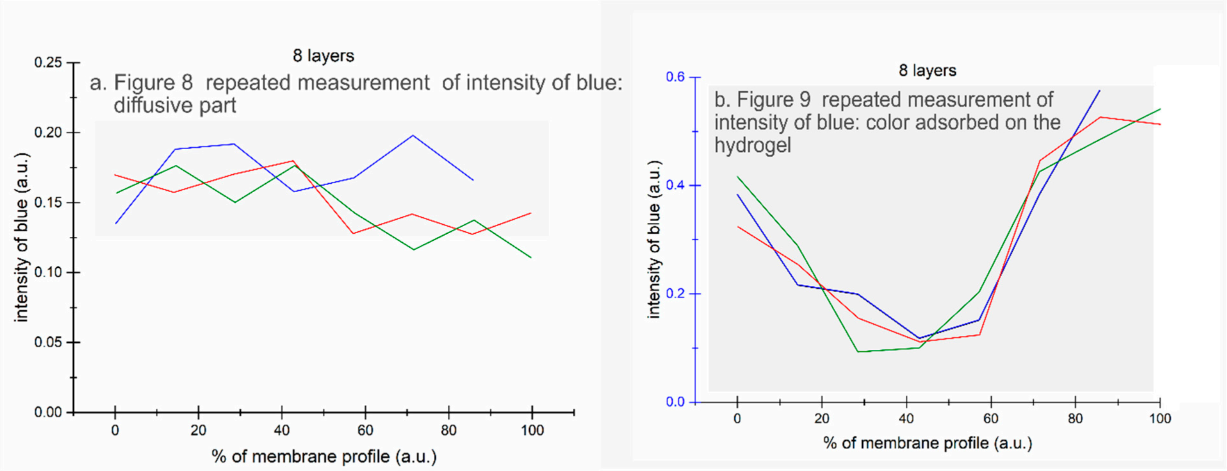

Figure S1: intensity of blue for three samples with 8 layers a. Main text - Figure 8b, b. Main text - Figure 9b.
